# Bibliographical

**Published:** 1888-02

**Authors:** 


### BIBLIOGRAPHICAL.

The Evolution of Immortality; or Suggestion of an Individual Immortality based upon Our Organic and Life History. By C. T. Stockwell. Chicago: Charles H. Kerr & Co. 1887. Price, $1.00.

This is a fascinating little volume, and places our good friend, Dr. Stockwell, in a new light. He is earnest and honest in whatever he says and does, his premises are always clearly stated, and in this volume he proves that he is a metaphysician of no mean acquirements. A perusal of this work will astonish those who have known only his professional and scientific life, for they will view him from another and favorable standpoint. Yet his many friends will recognize the same mental characteristics that are exhibited in his professional writings, for he impresses his own genial personality upon all the products of his pen. For this reason it is a difficult task for a personal friend and admirer impartially to review the book. It is so hard to dissociate it from its author and to prevent personal regard from improperly biasing the mind in estimating the book.

While we read with continued pleasure it was not always with approval of the reasoning. The book is a singular compound of the dead past with the living present—of the theories which were the outcome of the ages of darkness, and the scientific verities of today—of the conception of creation and life as taught us by those who claimed to have a direct revelation from above, but which was always the reflection of the views and beliefs of those through whom the revelation was given, and of the unerring truth as written in the imperishable works of creation by the very finger of the Creator himself. And yet so ingeniously are they woven together into the fabric of the book that it is hard to say just where a protest could begin. Like most metaphysicians, Dr. Stockwell begins where he should leave off—that is, with a theory. There is sometimes perceptible a curious confounding of scientific facts, and a singular translation of technical terms. For instance, force is sometimes alluded to as a mode of motion, and again as an entity. Inheritance surely is not a force; it is simply inertia—resistance to active differentiation. Adaptation to environment is but the friction on which force is too often wasted. Of *spiritual* force we can have no real conception, and hence it exists for us but as a name. When it shall have been dynamically demonstrated we may take its influence into account, but scientists have no right to employ unknown and undemonstrable qualities. The same influence that brings about the rupture of the Graafian vesicle bursts the germinal point in the kernel of corn, and hence, if there be a body and a spirit in the one, there should be in the other.

But while the book may be open to criticism from a scientific point of view, metaphysically it is charming, through its internal evidence of profound thought and its fertile suggestiveness. There is so much of ingenuity displayed in the argument, and old facts are presented in such new lights, there is such a vraisemblance of demonstrated things in its speculative reasonings, there are so many mere eidolons which are fully personified, that all of Dr. Stockwell's friends should desire to purchase and read the book, if it be only as a souvenir of the man; and if all his friends do order it, a new edition will be called for within a week. It may be obtained by sending one dollar to him at Springfield, Mass.

A MEMORIAL OF MEDICAL JURISPRUDENCE; with Special Reference to Diseases of the Nervous System. By ALLEN McLANE HAMILTON, M. D. Illustrated. 1887. E. B. Treat, 771 Broadway. Price, $2.75.

This is another of the series of volumes published by Mr. Treat, under the name of "Medical Classics." It is intended as a book of reference for lawyers and doctors. It is not a treatise upon forensic medicine, but rather a hand-book of the conditions and disturbances of the nervous system that are so often the foundation for suits at law, or which are involved in the settlement of the estates of deceased persons. The different chapters treat of insanity and its medico-legal relations; hysterical conditions and feigned diseases; epilepsy; alcoholism; suicide; cranial and spinal injuries. Each of these chapters is subdivided and considers its subject under exceedingly well classified sub-heads, with reports of cases, legal decisions, etc. There is a wealth of information in the book which every medical practitioner might study with profit; not alone of the legal bearings of neural and other disturbances, but of their pathology and clinical aspects as well, for the work was written by a medical man, and not by a lawyer.

NOTE.—The remainder of "Bibliographical" is crowded out of this number.

